# Cytotoxic Activity of Organotin(IV) Derivatives with Triazolopyrimidine Containing Exocyclic Oxygen Atoms

**DOI:** 10.3390/molecules25040859

**Published:** 2020-02-15

**Authors:** Alessandro Attanzio, Simone D’Agostino, Rosalia Busà, Anna Frazzitta, Simona Rubino, Maria Assunta Girasolo, Piera Sabatino, Luisa Tesoriere

**Affiliations:** 1 Department of Biological, Chemical and Pharmaceutical Sciences and Technologies (STEBICEF), University of Palermo, Parco d’Orleans II, Viale delle Scienze-Pad., 16-90128 Palermo, Italy; alessandro.attanzio@unipa.it (A.A.); rosalia.busa@unipa.it (R.B.); anna.frazzitta@unipa.it (A.F.); simona.rubino@unipa.it (S.R.); assunta.girasolo@unipa.it (M.A.G.); 2 Department of Chemistry “G. Ciamician”, University of Bologna, via F. Selmi 2, 40126 Bologna, Italy; simone.dagostino2@unibo.it

**Keywords:** triazolopyrimidine, organotin(IV), apoptosis, in vitro anticancer activity, crystal structure, metallodrugs

## Abstract

In this study cytotoxicity of organotin(IV) compounds with 1,2,4-triazolo[1,5-*a*]pyrimidines, Me_3_Sn(5tpO) (**1**), n-Bu_3_Sn(5tpO) (**2**), Me_3_Sn(mtpO) (**3**), n-Bu_3_Sn(mtpO) (**4**), n-Bu_3_Sn(HtpO_2_) (**5**), Ph_3_Sn(HtpO_2_) (**6**) where **5HtpO** = 4,5-dihydro-5-oxo-[1,2,4]triazolo-[1,5-*a*]pyrimidine, **HmtpO** = 4,7-dihydro-5-methyl-7-oxo-[1,2,4]triazolo-[1,5-*a*]pyrimidine, and **H_2_tpO_2_** = 4,5,6,7-tetrahydro-5,7- dioxo-[1,2,4]triazolo-[1,5-*a*]-pyrimidine, was assessed on three different human tumor cell lines: HCT-116 (colorectal carcinoma), HepG2 (hepatocarcinoma) and MCF-7 (breast cancer). While **1** and **3** were inactive, compounds **2**, **4**, **5** and **6** inhibited the growth of the three tumor cell lines with IC_50_ values in the submicromolar range and showed high selectivity indexes towards the tumor cells (SI > 90). The mechanism of cell death triggered by the organotin(IV) derivatives, investigated on HCT-116 cells, was apoptotic, as evident from the externalization of phosphatidylserine to the cell surface, and occurred via the intrinsic pathway with fall of mitochondrial inner membrane potential and production of reactive oxygen species. While compound **6** arrested the cell progression in the G2/M cell cycle phase and increased p53 and p21 levels, compounds **2**, **4** and **5** blocked cell duplication in the G1 phase without affecting the expression of either of the two tumor suppressor proteins. Compounds **1** and **2** were also investigated using single crystal X-ray diffraction and found to be, in both cases, coordination polymers forming 1 D chains based on metal-ligand interactions. Interestingly, for n-Bu_3_Sn(5tpO)(**2**) H-bonding interactions between 5tpO^−^ ligands belonging to adjacent chains were also detected that resemble the “base-pairing” assembly and could be responsible for the higher biological activity compared to compound **1**. In addition, they are the first example of bidentate N(3), O coordination for the 5HtpO ligand on two adjacent metal atoms.

## 1. Introduction

Organotin (IV) compounds have been receiving increasing attention over the past years [1,2,3,4,5,6] due to their possible use as biologically active non-platinum chemotherapeutic agents, that might have lower toxicity, better excretion properties and fewer side effects than platinum based drugs [7,8,9]. These compounds, even at low doses, present high anti-proliferative activity which, in some cases, is higher than that of cisplatin. Organotin compounds induce apoptosis by binding to DNA at external phosphate groups and by altering the intracellular metabolism of the phospholipids. Investigations have been carried out to test their antitumor activity and it has been observed that several triorganotin derivatives show very promising in vitro antitumor properties against a wide panel of tumor cell lines of human origin unlike di- and mono-organotin (IV) analogs, due to their ability to bind proteins [10]. 

1,2,4-triazolo[1,5-*a*] pyrimidine derivatives have been used as model systems to mimic the reactivity between purines and several metal ions [11] due to their ability to coordinate through different nitrogen atoms with accessible electron pairs in their basic skeleton. Furthermore, their versatility can be increased by changing the number of exocyclic substituents on the triazolopyrimidine rings. Most of the significant investigations with coordination compounds have been directed to the development of anti-tumor compounds of different metals [12] that could show improved therapeutic indexes and wider activity spectra. Such derivatives are interesting for their many biological effects such as antimicrobial [13,14], antitumor [15,16,17,18,19] and antiparasitic [20,21,22] activity.

The ligand 4,5-dihydro-5-oxo-[1,2,4]triazolo-[1,5-*a*] pyrimidine (5HtpO), the structures of which were described by Abul Haj et al. [23], is a triazolopyrimidine containing exocyclic oxygen atoms and is an isomer of the purine base hypoxanthine. Various studies have shown that the use of additional positions of coordination for oxygen with complexing capacity and the presence of a metal ion improve the antiparasitic [24] and antibacterial [25] activities. 

The 4,7-dihydro-5-methyl-7-oxo[1,2,4]triazolo[1,5-*a*] pyrimidine (HmtpO) can also be considered an analogue of the natural occurring nucleobase hypoxanthine [26]. The structure of the ligand raises substantial interest due to its unique biological and medical potential. In *cis*–*trans*-[PtCl_2_(OH)_2_(NH_3_)(HmtpO)], the HmtpO is coordinated through the N3 nitrogen atom in a monodentate fashion [27]. Moreover, *cis*–*trans*-[PtCl_2_(OH)_2_(NH_3_)(HmtpO)] showed highly promising cytotoxicity in vitro against human acute promyelocytic leukemia (HL60). In the monomeric complex [Cu(HmtpO)_2_(H_2_O)_3_] (ClO_4_)_2_∙H_2_O, the heterocyclic ligands show both N3 monodentate and N1, O71 bidentate modes of coordination. However, in the bidimensional compound {[Cu(HmtpO)_2_(H_2_O)_2_] (ClO_4_)_2_∙2HmtpO}_n_, a unique N3, O71 bidentate bridging coordination mode of the HmtpO ligands was observed. A third interesting tridentate bridging coordination pattern of HmtpO by the nitrogen atoms N1 and N3 and the oxygen atom O71 was observed in 1D chains of {[Co(HmtpO)(H_2_O)_3_] (ClO_4_)_2_∙2H_2_O}_n_ [28]. 

The other triazolopyrimidine containing exocyclic oxygen atoms is 4,5,6,7-tetrahydro-5,7-dioxo[1,2,4]-triazolo[1,5-*a*] pyrimidine (H_2_tpO_2_), described by Orihuela et al. [29], which is also an isomer of xanthine. Only a few complexes of this ligand are reported in the literature, such as those with the divalent transition metals of the first transition series from Mn to Zn [30].

**Scheme 1:**
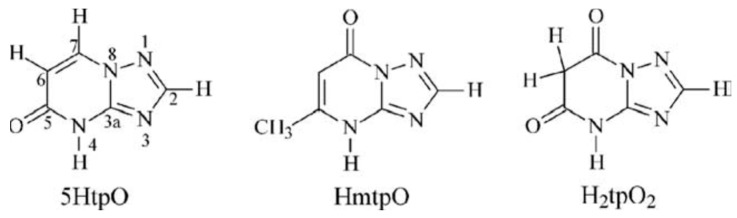
The chemical structures of the ligands employed and the atom numbering scheme [25].

To further explore the influence of triazolopyrimidine with exocyclic oxygen atoms and possible structure-activity relationships, we decided to study the biological properties of organotin(IV) coordination compounds with 5HtpO, HmtpO and H_2_tpO_2_. Scheme 1 displays the oxo-derivatives indicating the International Union of Pure and Applied Chemistry (IUPAC) numbering scheme used for these heterocycles. In a previous article [25], we reported the synthesis and the characterization of the triazolopyrimidine tin(IV) derivatives Me_3_Sn(5tpO) (**1**), n-Bu_3_Sn(5tpO) (**2**), Me_3_Sn(mtpO) (**3**), n-Bu_3_Sn(mtpO) (**4**), n-Bu_3_Sn(HtpO_2_) (**5**), Ph_3_Sn(HtpO_2_) (**6**), which were also preliminarily tested in vitro for antimicrobial activity. As a continuation of our work, in this study we present the crystal structures of compounds Me_3_Sn(5tpO) **1** and n-Bu_3_Sn(5tpO) **2** and assess the cytotoxic activity of the compounds **1**-**6** on a number of human carcinoma cell lines to evaluate the influence of the substituents connected to the triazolopyrimidine ring on the antitumor activity. All compounds were evaluated for their in vitro anti-proliferative activity against HCT-116 (human colorectal carcinoma), HepG2 (human hepatocellular cancer) and MCF-7 (human breast cancer) cells, while selective anti-tumor activity was estimated on intestinal normal-like differentiated Caco-2 (human colorectal carcinoma) cells and human bronchial epithelial cells (16-HBE). The cytotoxic mechanism of the more active compounds was investigated on HCT-116 cells. 

## 2. Results and Discussion

### 2.1. Crystal Structure of Me_3_Sn(5tpO), ***1*** and n-Bu_3_Sn(5tpO), ***2***

**Scheme 2:**
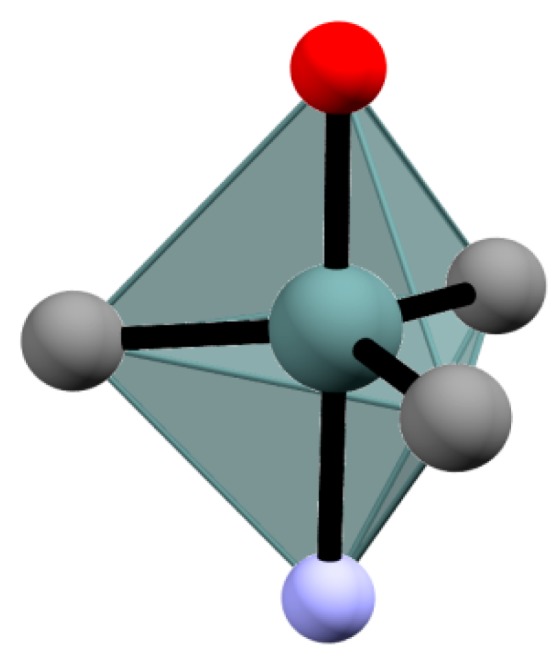
Coordination environment of pentacoordinated Sn (IV) in Me_3_Sn(5tpO) **1** and n-Bu_3_Sn(5tpO) **2.**

**Figure 1:**
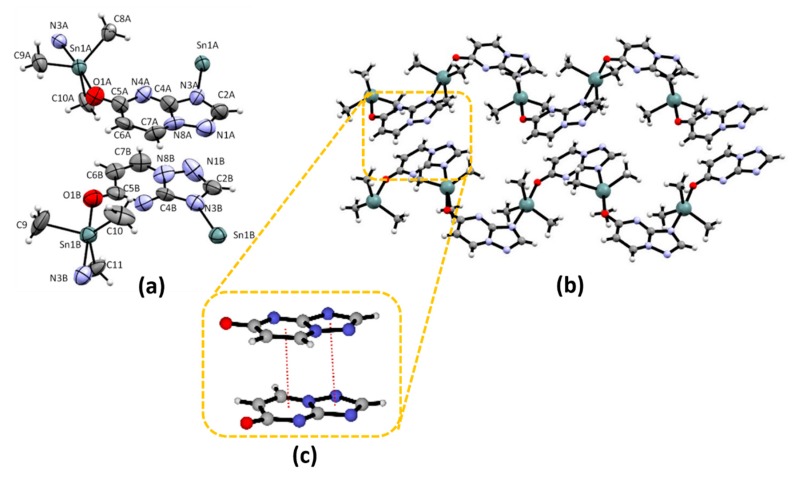
(**a**) The asymmetric unit for **1**, showing the atomic crystallographic numbering scheme; displacement ellipsoids are drawn at the 50% probability level. (**b**) View of the 1D chains. (**c**) π-π stacking interactions detected between ligands belonging to adjacent chains.

**Figure 2:**
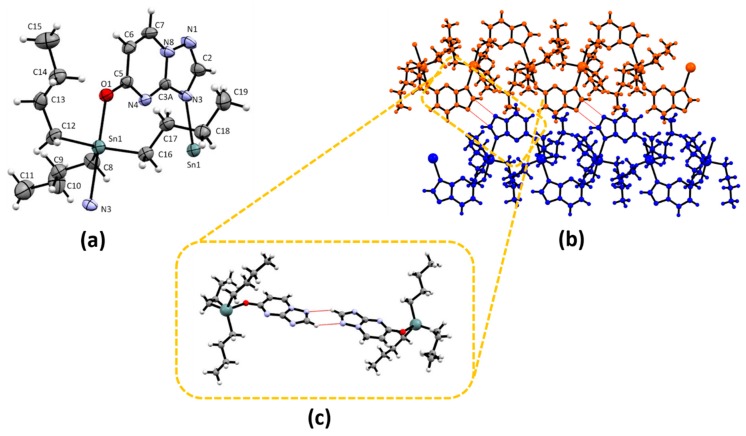
(**a**) The asymmetric unit for **2**, showing the atomic crystallographic numbering scheme; displacement ellipsoids are drawn at the 50% probability level. (**b**) View of two adjacent 1D chains. (**c**) Detail of the “base-pairing like” inter-catena H-bond interaction.

Despite several attempts under different crystallization conditions, only compounds Me_3_Sn(5tpO) **1** and n-Bu_3_Sn(5tpO) **2** gave suitable crystals for single crystal X-ray diffraction, SCXRD, investigations. Their crystal structures are given in Figure 1 and Figure 2, respectively, together with their crystallographic numbering. Both compounds feature a pentacoordinated tin metal in a distorted trigonal bypyramid geometry (see Scheme 2) formed by two crystallographically equivalent oxo-tp ligands occupying the apical positions and by the methyl groups in **1** and the n-butyl ones in **2** occupying the trigonal plane. Apical positions are invariably held by the tp N(3) atom on one side and the exocyclic O atom, belonging to the symmetry-related oxo-tp ligand, on the other side, [N(3)-Sn-O angles = 176.7 (4) ave. for **1** and 176.9(1)° for **2**], thus generating a one-dimensional polymeric chain along the b-axis direction, in agreement with previous Mössbauer data [25]. This kind of bidentate bridging N, O coordination is, to the best of our knowledge, quite unusual for this oxo-ligand; actually, the crystal structures of only few coordination compounds of this ligand with transition metals are known. One of them shows monodentate coordination through N(3) for the octahedral Ni atom, as for the unsubstituted tp ligand, in trans-diaqua-bis(4,5-dihydro-1,2,4-triazolo[2,3-*a*] pyrimidin-5-one-N3)-bis-isothiocyanato)-nickel(II) [31]. Bidentation at N(3), N(4) is used, on the other hand, in homo-dinuclear compounds where they function as a bridge on two metal atoms, such as Ag, in bis(µ^2^-4,5,dihydro-5-oxo[1,2,4]triazolo[1,5-*a*]pyrimidine-di-silver(I)dinitrato and bis(µ^2^-4,5,dihydro-5-oxo[1,2,4]triazolo[1,5-*a*]pyrimidine-di-silver(i)diperchlorate dehydrate [32], as Pd, in bis(µ^2^-4,5,dihydro-5-oxo[1,2,4]triazolo[1,5-*a*pyrimidine-5-one-*N,N*′-(1-10-phenantroline) palladium(II) dinitrate tetrahydrate [33], or Cu, in bis (µ^2^-[1,2,4]triazolo[1,5-*a*]pyrimidine-5-onato)-bis(m2-hydrogensuccinato)-diaqua-di-copper(II) [31] compounds. In the polynuclear [Cd_2_(µ-5tpO)_2_(µ_4_-suc)(H_2_O)_2_]_n_ [34], on the other hand, due to the eptacoordination of the metal, three out of the four possible electron-donor groups, i.e., N(3), N(4) and O, of the 5tpO^−^ ligand are used in a tridentate ligand coordination bridging on two adjacent metal atoms, making it the first example of involvement in metal ligation of the O atom belonging to the oxo moiety. Our compounds are then the first examples of bidentate N(3), O coordination bridging two adjacent metal atoms in homo-polymeric Sn catena compounds.

Compound **1** exhibits two molecules, A and B, in the asymmetric unit. The two molecules each form a metal coordination polymer, and π-π stacking interactions link the polymers into a three-dimensional network (with contacts ranging around 3.4 Å, Figure 1b,c). The Sn∙∙∙Sn interactions are slightly shorter in A (5.70 Å) than in B molecules (5.74 Å), suggesting closer ligands packing for the less encumbered tin ligands with respect to compound **2**. Compound **2,** as well, features a polymeric chain with Sn∙∙∙Sn interactions of 6 Å, but differently from compound **1**, it is characterized by “base-pairing like” inter-strands double H-bond interactions from C(2)-H(2) to N(1) and, vice versa, from the triazole moieties of symmetry-related molecules (Figure 2b,c). With a donor-acceptor distance of 3.18 Å and D-H∙∙∙A angle of 130°, this DNA-mimicking behavior between adjacent chains, somewhat predicted for nucleobase analogues such as our oxo-tp ligands but not so commonly shown, seems to dictate the molecular packing of **2.** Actually, no aromatic interactions take place. Selected geometric parameters (Å, °) for **2** are summarized in Table 1.

### 2.2. Biology 

#### 2.2.1. Cytotoxicity Assay 

**Figure 3:**
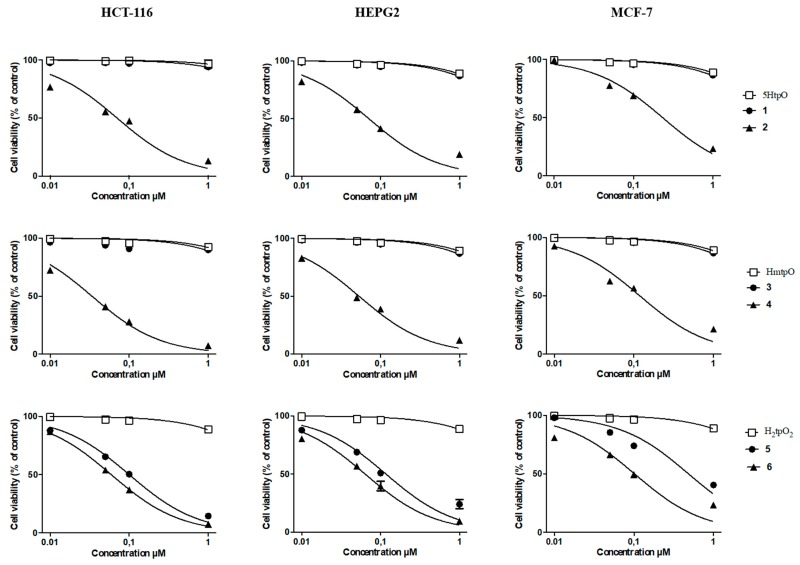
Effect of **1–6** and their ligands on the viability of HCT-116 (human colorectal carcinoma), HepG2 (human hepatocellular cancer) and MCF-7 (human breast cancer) cells after 24 h treatment. Cells were treated with compounds **1**–**6** and cell survival was measured using MTT assay in comparison to cells treated with vehicles alone (control), as reported in methods. Values are the mean ± SD of three separate experiments carried out in triplicate.

Cytotoxicity of the organotin(IV) compounds **1**–**6**, characterized by three different ligands **5HtpO**, **HmtpO** and **H_2_tpO_2_**, was studied on human tumor cell lines HCT-116, HepG2 and MCF-7 using a MTT test. The viability of cellular monolayers after 24 h of treatment with the compounds and their ligands, tested in the 0.01–1 µM range, is reported in Figure 3. While all the ligands and the compounds Me_3_Sn(5tpO) **1** and Me_3_Sn(mtpO) **3** had no influence on cell growth under the conditions used, compounds n-Bu_3_Sn(5tpO) **2**, n-Bu_3_Sn(mtpO) **4**, n-Bu_3_Sn(HtpO_2_) **5** and Ph_3_Sn(HtpO_2_) **6** effectively inhibited cell viability in a dose-dependent manner. Based on the IC_50_ values, the cytotoxicity of the compounds on HCT-116 and HepG2 cells followed the order **4** > **6** > **2** > **5**, while on MCF-7 cells the compounds appeared less cytotoxic following the order **6** > **4** > **2** > **5** (Table 2). Moreover, complexes **2**, **4**, **5** showed a different cytotoxic activity related to their ligands being **HmtpO** > **5HtpO** > **H_2_tpO_2_**. Ph_3_Sn(HtpO_2_) **6** appeared more effective against the growth of all the cell lines than n-Bu_3_Sn(HtpO_2_) **5**, which has the same ligand. Interestingly, the compounds were more cytotoxic than cisplatin, used for comparison as gold standard (Table 2).

**Figure 4:**
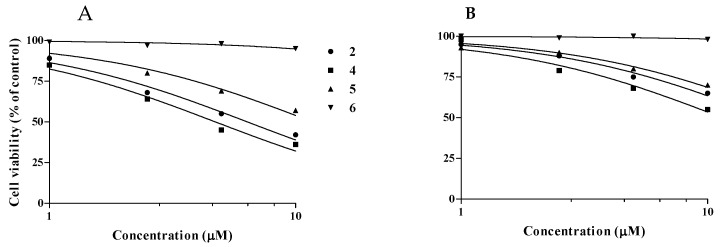
Effect of compounds **2**, **4**, **5** and **6** on the viability of human intestinal normal-like differentiated Caco-2 (human colorectal carcinoma) (**A**) or 16-HBE (human bronchial epithelial) cells (**B**). Cells were treated with the compounds and cell viability was measured after 24 h using MTT assay in comparison to cells treated with vehicle alone (control). Values are the mean ± SD of three separate experiments in triplicate.

Selective cytotoxicity is a pivotal requirement for anticancer drugs, and activity of the compounds on intestinal normal-like differentiated Caco-2 cells and human bronchial epithelial cells (16-HBE) was assessed in additional experiments. While a reduction of the cell survival was detected after treatment with the **2**, **4** and **5** derivatives in the 1–10 μM range on both the cell lines, compound **6** did not affect the cell viability at any assayed concentration (Figure 4). However, as the calculated selectivity index (SI, Table 3) greatly exceeds the value accepted as threshold for antitumor drugs (SI = 2.0) [35], potential use of all the synthesized complexes as antiproliferative agents could be considered.

#### 2.2.2. Cell Death

**Figure 5:**
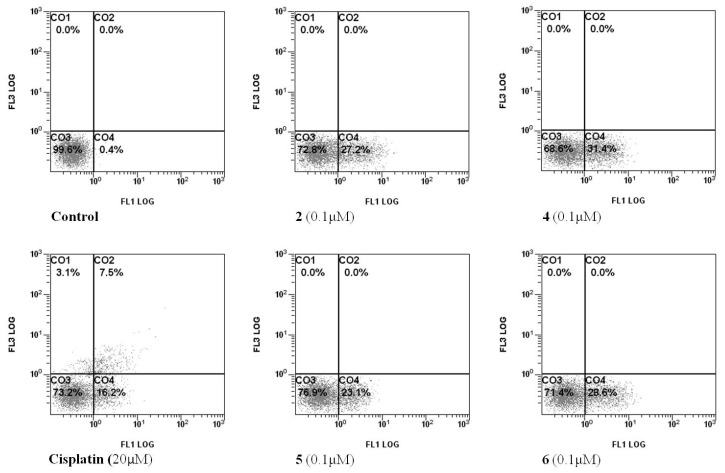
Flow cytometric analysis for the quantification, using AnnexinV/PI double staining, of **2**, **4**, **5** and **6** induced apoptosis in HCT-116 cells. Cell monolayers were incubated for 24 h in the absence (control) or in the presence of the individual compounds or cisplatin and submitted to double staining with Annexin V/PI as reported in Methods. CO3, viable cells (AnnexinV−/PI−); CO4, cells in early apoptosis (AnnexinV+/PI−); CO2, cells in late apoptosis (AnnexinV+/PI+); CO1, necrotic cells (AnnexinV−/PI+). Representative images of three experiments with comparable results.

In order to analyze the mode of cell death induced by the active organotin compounds, HCT-116 were treated with the individual compounds at 0.1 µM concentration for 24 h before double-staining with AnnexinV/PI. Cisplatin, one of the most widely used chemotherapy drugs, was used as positive control. The results showed that all tested compounds increased the number of early apoptotic dead HCT-116 cells without causing any necrotic damage, generally accompanied in vivo by inflammatory processes (Figure 5).

#### 2.2.3. Mitochondrial Dysfunction

**Figure 6:**
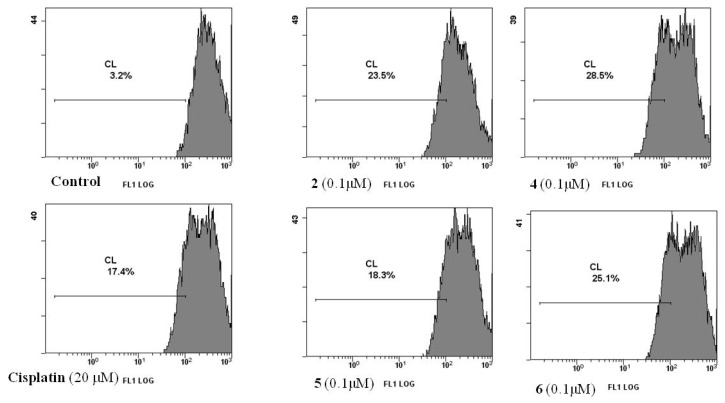
Effects of **2**, **4**, **5** and **6** on dissipation of mitochondrial transmembrane potential (Δψm) in HCT-116 cells. After treatment with the compounds for 24 h, cells were stained with the lipophilic cationic fluorochrome DiOC6(3) and flow cytometry analysis was carried out as reported in Methods. Cisplatin was used as positive control. Representative images of three experiments with comparable results.

In many systems, apoptosis is associated with the loss of mitochondrial inner membrane potential (Δψm), which is responsible for the release of some pro-apoptotic factors from the organelle. We investigated the involvement of mitochondria in apoptosis induced by the organotin compounds using the lipophilic cationic fluorochrome DiOC6, a fluorescent mitochondria-specific and voltage-dependent dye. Measurements of Δψm using flow cytometry analysis showed that compounds **2**, **4**, **5** and **6** at 0.1 µM induced in HCT-116 cells, after 24 h of treatment, a significant decrease in DiOC6(3) uptake, indicating induction of mitochondrial dysfunction (Figure 6). Mitochondrial permeability dysfunction appeared comparable to that produced by 20 µM cisplatin (Figure 6).

**Figure 7:**
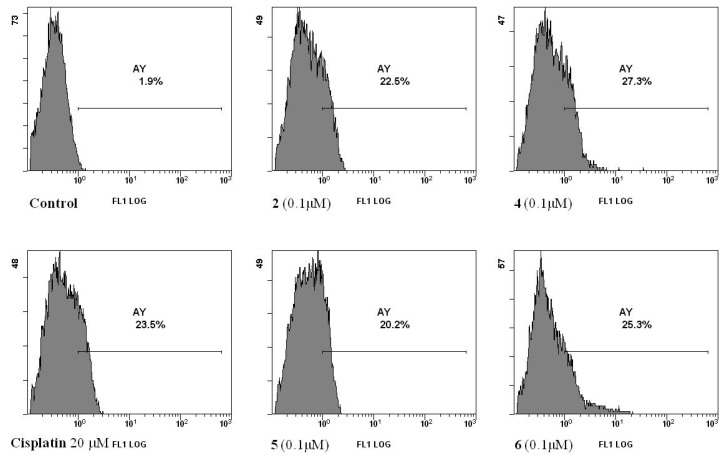
Effect of compounds **2**, **4**, **5** and **6** on the production of ROS in HCT-116 cells. ROS were detected using fluorescence intensity of 2′,7′-dichlorofluorescin diacetate-stained cells, as determined using flow cytometry after 24 h treatment with the compounds. Control, cells treated with vehicle. Cisplatin was used as positive control. Representative images of three experiments with comparable results.

Since the mitochondrial dysfunction may be related to intracellular redox unbalance and induction of apoptosis in various cell types, we explored whether the organotin(IV) complexes could stimulate ROS generation in HCT-116 cells. As shown in Figure 7, compounds **2**, **4**, **5** and **6** after 24 h of treatment induced, when compared to control, an increase in ROS production, cytofluorimetrically detected by the fluorescent dye DCFH-DA. Cisplatin, at 20 µM, produced a similar effect.

#### 2.2.4. Cell Cycle Analysis

**Figure 8:**
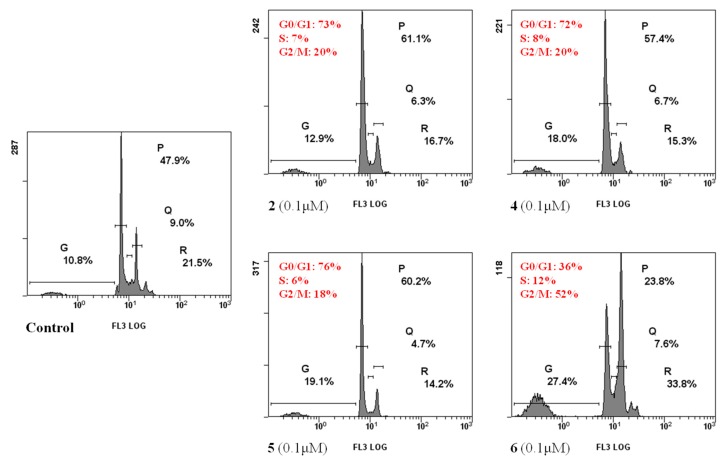
Effect of compounds **2**, **4**, **5** and **6** on cell cycle distribution of HCT-116 cells. Flow cytometric analysis of propidium iodide-stained cells after 24 h treatment with the compounds. The percentage of cells in the different phases of the cycle was calculated using Expo32 software. Percentage of viable cells in the different phases are given in each picture. Representative images of three experiments with comparable results.

A disturbance of the cellular redox state could induce apoptosis triggering a ROS-dependent signaling pathway leading to block of the cell cycle. The effects of active **2**, **4**, **5** and **6** complexes on HCT-116 cell cycle distribution were determined using flow cytometric analysis after staining of DNA with propidium iodide (PI). As shown in Figure 8, compounds **2**, **4** and **5** after 24 h of treatment induced a block of cell duplication in the G0/G1 phase, whereas compound **6** caused a cell cycle arrest in phase G2/M. Although compounds **5** and **6** share the same ligand (H_2_tpO_2_), they caused proliferation arrest at different phases of the cell cycle, underlining the relevance of the organotin moieties in the biological mechanism of the complexes. In the end, all compounds caused an increase of the percentage of cells in the sub-G1 phase, which is representative of cells with fragmented DNA, confirming that the cells were carried through apoptosis.

#### 2.2.5. p53-p21^WAF1^ Protein Levels

**Figure 9:**
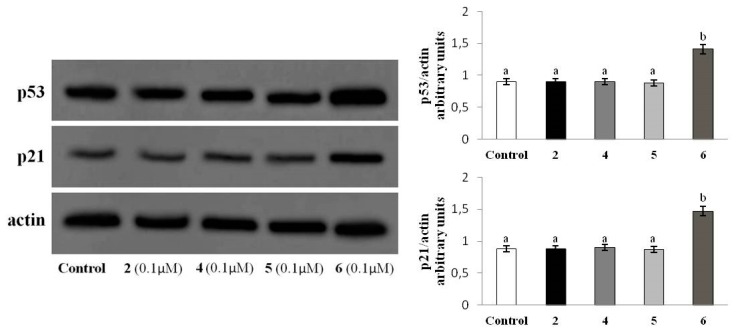
p53 and p21^WAF1^ levels using immunoblotting with densitometric analysis of the immunoblots. Representative images of three experiments with comparable results. Data of the densitometric analysis are the mean ± SD. Bars with different letters are significantly different with *p* < 0.05 (ANOVA associated with Tukey test).

p53, a major tumor suppressor protein, orchestrates various biological events including cell cycle arrest, cellular senescence and DNA repair, in addition to apoptosis [36]. Functionally, p53 is a transcription factor forming a homo-tetramer to activate nearly 500 target genes [37]. In mammalian cells, p53 induces a large number of genes involved in various steps of apoptosis signaling and execution [38]. These include BH3 domain-only proapoptotic proteins, death receptors and apoptosis execution factors. In addition, p53 promotes the transcriptional activation of p21^WAF1^ that, binding to several cyclins, causes arrest of the cell cycle at different phases [39,40]. To assess the involvement of p53-p21^WAF1^ signaling pathway in the apoptosis triggered by the organotin compounds, we investigated the compounds’ influence on the expression of both the proteins by immunoblotting. While 24 h treatment of HCT 116 cells with **2**, **4**, **5** did not vary the level of p53 or p21, incubation with compound **6** resulted in a net increment (about 1, 5fold) of the levels of both the tumor suppressor proteins (Figure 9).

## 3. Materials and Methods

### 3.1. Synthesis of Triorganotin(IV) Compounds 

The synthesis and characterization of the compounds Me_3_Sn(5tpO) (**1**), n-Bu_3_Sn(5tpO) (**2**), Me_3_Sn(mtpO) (**3**), n-Bu_3_Sn(mtpO) (**4**), n-Bu_3_Sn(HtpO_2_) (**5**), Ph_3_Sn(HtpO_2_) (**6**) (where **5HtpO** = 4,5-dihydro-5-oxo-[1,2,4]triazolo-[1,5-*a*]pyrimidine, **HmtpO** = 4,7-dihydro-5-methyl-7-oxo-[1,2,4] triazolo-[1,5-*a*]pyrimidine, and **H_2_tpO_2_** = 4,5,6,7-tetrahydro-5,7-dioxo-[1,2,4]triazolo-[1,5-*a*]- pyrimidine), were carried out according to the method reported in our previous study [25] and will not be discussed here. 

### 3.2. X-ray Crystallography of Me_3_Sn(5tpO), ***1*** and n-Bu_3_Sn(5tpO), ***2***

Single crystal data for compounds Me_3_Sn(5tpO) **1** and n-Bu_3_Sn(5tpO) **2** were collected at low temperature (200 K) on an Oxford XCalibur S CCD diffractometer equipped with a graphite monochromator (Mo-Kα radiation, λ = 0.71073Å) and with a cryostat CryoStream800. Two twin unit cells were indexed for **1** and **2**, and the reflection data were integrated with the default configuration for twinned crystals of CrysAlisPro software. Subsequent structure solution and refinement were performed using the HKLF4 file containing non-overlapped reflections. All non-hydrogen atoms were refined anisotropically. H_CH_ atoms for all compounds were added in calculated positions and refined riding on their respective carbon atoms. SHELX97 [41] was used for structure solution and refinement on F2. The program Mercury [42] was used to calculate intermolecular interactions and for molecular graphics. Data collection and refinement details are listed in Table 4. Crystal data can be obtained free of charge via www.ccdc.cam.ac.uk/conts/retrieving.html (or from the Cambridge Crystallographic Data Centre, 12 Union Road, Cambridge CB21EZ, UK; fax: (+44)1223-336-033; or e-mail: deposit@ccdc.cam.ac.uk). CCDC numbers 1908619-1908620.

### 3.3. Biological Studies

#### 3.3.1. Viability Assay 

The synthesized compounds **1**–**6** with three different ligands **5HtpO**, **HmtpO** and **H_2_tpO_2_** were dissolved in dimethylsulfoxide (DMSO) and then diluted in culture medium so that the effective DMSO concentration did not exceed 0.1%. HTC116 (human colorectal carcinoma), HepG2 (human hepatocellular carcinoma), MCF-7 (human breast cancer), 16-HBE (human bronchial epithelium) and Caco-2 (human colorectal carcinoma) cell lines were purchased from American Type Culture Collection, Rockville, MD, USA. All cell lines were grown in RPMI medium supplemented with L-glutamine (2 mM), 10% fetal bovine serum (FBS), penicillin (100 U/mL), streptomycin (100 µg/mL) and gentamicin (5 μg/mL). Cells were maintained in log phase by seeding twice a week at a density of 3 × 10^8^ cells/L in humidified 5% CO_2_ atmosphere, at 37 °C. In all experiments, after plating, cells were allowed to adhere overnight and then treated with the compounds or vehicle alone (control cells), whereas Caco-2 cells were treated 15 days after confluence, at which time the cells are differentiated in normal intestinal-like cells [43]. No differences were found between cells treated with DMSO 0.1% and untreated cells in terms of cell number and viability.

Cytotoxic activity of the organotin compounds against human tumor cell lines (HCT-116, HepG2 and MCF-7) and human normal cells (16-HBE and intestinal-like differentiated Caco2 cells) was determined using the MTT colorimetric assay based on the reduction of 3-(4,5-dimethyl-2-thiazolyl)bromide-2,5-diphenyl-2H-tetrazolium (MTT) to purple formazan using mitochondrial dehydrogenases of living cells, as reported [44]. This method is commonly used to illustrate inhibition of cellular proliferation. Monolayer cultures were treated for 24 h with various concentrations (0.01-1 µM) of the drugs. Cisplatin was used for comparison. Briefly, all cell lines were seeded at 1.0 × 10^4^ cells/well in 96-well plates containing 200 μL RPMI. When appropriated, cells were washed with fresh medium and incubated with the compounds in RPMI. After incubation, cells were washed, and 50 μL FBS-free medium containing 5 mg/mL MTT was added. The medium was discarded after a 2 h incubation at 37 °C by centrifugation, and formazan blue formed in the cells was dissolved in DMSO. The absorbance, measured at 570 nm in a microplate reader (Bio-RAD, Hercules, CA, USA) of MTT formazan of control cells was taken as 100% of viability. The growth inhibition activity of compounds was estimated by comparison with the not-treated control. The values for the inhibitory concentration for 50% of the tested compounds (IC_50_) were calculated using a sigmoidal model using GraphPad Prism 5.02 from GraphPad Software (San Diego, CA, USA). Each experiment was repeated three times in triplicate to obtain the mean values.

#### 3.3.2. Selectivity Index (SI)

Formazan of control cells was taken as 100% viability and the 50% lethal concentrations of the synthesized complexes (LC_50_) were determined by plotting the graph of cell viability versus the concentrations. The selectivity index (SI) values were calculated for each compound by dividing the LC_50_ of normal differentiated Caco2 or 16-HBE cells by the IC_50_ of HCT-116 tumor cells. Each experiment was repeated three times in triplicate.

#### 3.3.3. Measurement of Phosphatidylserine Exposure 

The externalization of phosphatidylserine to the cell surface was detected using flow cytometry by double staining with annexin V/PI. Tumor cells were seeded in triplicate in 24-well culture plates at a density of 5.0 × 10^4^ cells/cm^2^. After an overnight incubation, cells were washed with fresh medium and incubated with the compounds prepared as described above. After 24 h, cells were harvested by trypsinization and adjusted at 1.0 × 10^6^ cells/mL with buffer according to the manufacturer’s instructions (eBioscience, San Diego, CA, USA). One hundred μL of cell suspended solution was added to a new tube and incubated with 5 μL annexin V and 10 µL of a 20 µg/mL propidium iodide (PI) solution at room temperature in the dark for 15 min. Then samples of at least 1.0 × 10^4^ cells were subjected to fluorescence-activated cell sorting (FACS) analysis using Epics XL™ flow cytometer with Expo32 software (Beckman Coulter, Fullerton, CA, USA), using appropriate 2-bidimensional gating method.

#### 3.3.4. Measurement of Mitochondrial Transmembrane Potential 

Mitochondrial transmembrane potentials (Δψm) were assessed using flow cytofluorometry, with the cationic lipophilic dye 3,3′-dihexyloxacarbocyanine iodide(3) (DiOC6) (Molecular Probes, Inc., Life Technologies Italia, Monza, Italy), which accumulates in the mitochondrial matrix. Changes in mitochondrial membrane potential are indicated by a reduction in the DiOC6-induced fluorescence intensity. After 24 h treatment, cells were incubated with DiOC6 at a 40 nmol/L final concentration, for 15 min at 37 °C. After centrifugation, cells were washed with phosphate buffer saline (PBS) and suspended in 500 μL PBS. Then, samples of 1.0 × 10^4^ cells were subjected to FACS analysis.

#### 3.3.5. Measurement of Intracellular Reactive Oxygen Species (ROS)

ROS level was monitored by measuring fluorescence changes that resulted from intracellular oxidation of 2′,7′-dichlorofluorescin diacetate (DCFH-DA). DCFH-DA, at 10 μM final concentration, was added to the cell medium 30 min before the end of the treatment. The cells were collected using centrifugation for 5 min at 2000 rpm at 4 °C, washed, suspended in PBS and immediately subjected to FACS analysis. At least 1 × 10^4^ cells were analyzed for each sample.

#### 3.3.6. Cell Cycle Analysis

Cell cycle stage was analyzed using flow cytometry. Aliquots of 1.0 × 10^6^ cells were harvested using centrifugation, washed with PBS and incubated in the dark in a PBS solution containing Triton X100 (0.1%, *v*/*v*), 20 μg/mL propidium iodide (PI) and 200 µg/mL RNase, for 30 min, at room temperature. Then, samples were immediately subjected to FACS analysis. At least 1 × 10^4^ events were analyzed for each sample.

#### 3.3.7. Western Blot Analysis

After treatment with the compound, protein extracts were prepared and equal amounts of protein samples (80 μg/lane), subjected to SDS-PAGE, were transferred to nitrocellulose membrane as previously reported [45]. The immunoblot was incubated overnight at 4 °C with blocking solution (5% skim milk), followed by incubation with anti-p21 (F-5, Cat No SC-6246 Santa Cruz Biotechnology) or anti-p53 (DO-1, Cat No SC-126, Santa Cruz Biotechnology) for 1 h at room temperature. Blots were washed two times with Tween 20/Tris-buffered saline (TTBS) and incubated with a 1:2000 dilution of horseradish peroxidase (HRP)-conjugated anti-IgG antibody (Dako Denmark, Glostrup, Denmark) for 1 h at room temperature. Blots were again washed five times with TTBS and then developed using enhanced chemiluminescence (Amersham Life Science, Arlington Heights, IL, USA.). Immunoreactions were also performed using β-actin antibody as loading controls. 

#### 3.3.8. Statistical Analysis

Results are given as means and standard deviations. Unless stated otherwise, three independent observations were performed for each experiment thrice replicated. Calculations and graphs were obtained using the INSTAT-3 statistical software (GraphPad Software, Inc., San Diego, USA) with a test for normality followed by ANOVA, with Tukey’s correction for multiple comparisons. In all cases, significance was accepted if the null hypothesis was rejected at the *p* < 0.05 level.

## 4. Conclusions

The spectroscopic properties of compounds **1**–**6** have been previously reported [25]. In this study we report that compounds **2**, **4**, **5** and **6** effectively inhibited the cell growth of three different lines of human carcinoma (colon, liver and breast), with calculated submicromolar IC_50_ values. Cytotoxic activity of the compounds appeared 2‒3 orders of magnitude higher than that of cisplatin used as gold standard, and were highly selective (SI > 90) towards the tumor cells. Among the tri-n-butyltin(IV) derivatives **2**, **4** and **5**, the complex **4** with HmtpO showed stronger cytotoxicity. Although it has been previously demonstrated that n-Bu_3_Sn(HtpO_2_) **5** owns a greater antifungal and antibiofilm activity than Ph_3_Sn(HtpO_2_) **6** [25], the present study carried out on human tumor cells indicates an inverse antiproliferative efficacy.

The cell death mechanism exerted by the organotin(IV) derivatives was apoptosis, as established in HCT-116 cells using flow cytometric analysis of externalization of plasma membrane PS, and occurred via the intrinsic pathway, as ascertained using mitochondrial dysfunction. The signaling pathway of apoptosis induced by the compounds appeared distinct. In fact, while compound **6** arrested the cell progression in the G2/M cell cycle phase and increased p53 and p21^WAF1^ levels, compounds **2**, **4** and **5** induced a block of cell duplication in the G0/G1 phase without affecting either p53 or p21^WAF1^ expression. It is interesting in this regard to mention that a triphenyl derivative with *N*-*tert*-butoxycarbonyl-L-ornithine, previously synthesized and characterized in our laboratory, induced apoptosis in human hepatoma HepG2 cells associated with an increase of p53 levels [46], suggesting that the induction pathway of the oncoprotein is influenced by the Ph_3_Sn-moiety. At last, since p53 is defective in >50% of tumors, the ability of the **2**, **4** and **5** derivatives to induce apoptosis independently of p53 may offer an advantage in anti-tumor therapy. 

A SCXRD investigation was carried out on both compounds **1** and **2**, the only ones of the series to yield good enough crystals. As for compound **2**, its ability, through metal coordination, to form pairs of hydrogen bonded inter-strand, purine-like ligands may certainly be related to an enhanced propensity for recognition processes towards biological targets, in comparison to **1,** which is devoid of it and inactive. These ligand-mediated recognition processes may give a contribution towards organotin binding to DNA and help to potentially deliver new metal-based therapies to the clinic. Therefore, a further investigation on the detailed mechanism of pro-apoptotic action exerted specifically by compounds n-Bu_3_Sn(5tpO) **2** and n-Bu_3_Sn(mtpO) **4** may prove of great interest, and is actually planned in the near future, in order to better elucidate their specific signaling pathways.

## Figures and Tables

**Table 1:** Selected bond distances (Å) and angles (°) around the Sn(IV) ion.

	Me_3_Sn(5tpO) 1	n-Bu_3_Sn(5tpO) 2
Sn1···O1 (Å)	2.230(1)/2.310(1)	2.210(2)
Sn1···N3 (Å)	2.370(8)/2.348(8)	2.445(3)
Sn1···C (Å)	2.06(3)–2.17(2)	2.141(5)–2.152(5)
C=O (Å)	1.210(2)/1.241(2)	1.282(5)
O-Sn-N (°)	174.5(4)/178.9(5)	176.9(1)
Sn1···N3 (Å)	113.2(1)–129.1(1)	115.1(2)–123.3(2)
Sn1···C (Å)	84.3(6)–97.2(5)	91.7(1)–95.1(1)
C=O (Å)	86.8(7)–91.5(6)	83.7(1)–88.1(1)

**Table 2:** Calculated IC_50_ values of organotin(IV) compounds against the proliferation of different tumor cell lines.

Compounds	IC_50_ (nM ± SD)		
	HCT-116	HepG2	MCF-7
(**1**) Me_3_Sn(5tpO)	>1000	>1000	>1000
(**2**) n-Bu_3_Sn(5tpO)	70 ± 8	70 ± 6	233 ± 21
(**3**) Me_3_Sn(mtpO)	>1000	>1000	>1000
(**4**) n-Bu_3_Sn(mtpO)	34 ± 3	53 ± 6	118 ± 10
(**5**) n-Bu_3_Sn(HtpO_2_)	101 ± 11	117 ± 10	487 ± 47
(**6**) Ph_3_Sn(HtpO_2_)	60 ± 5	63 ± 5	102 ± 9
Cisplatin	46 × 10^3^ ± 3 × 10^3^	65 × 10^3^ ± 2 × 10^3^	10 × 10^3^ ± 1 × 10^3^

Values are the mean ± SD of three separate experiments in triplicate.

**Table 3:** Cytotoxic activity and selectivity index (SI) of the synthesized compounds against HCT-116 cancer cell line.

	Normal-Like Intestinal Cells LC_50_ (μM)	SI	16-HBE Cells LC_50_ (μM)	SI
**2**	6 ± 0.4	91	17 ± 1	240
**4**	5 ± 0.4	118	11 ± 1	323
**5**	12 ± 1	116	22 ± 1	217
**6**	184 ± 9	>1000	580 ± 22	>1000

Values are the mean ± SD of three separate experiments in triplicate. LC_50_ concentration, which is lethal to 50% of the normal cells, compared to untreated controls.

**Table 4:** Crystal data and refinement details for crystalline Me_3_Sn(5tpO) **1** and n-Bu_3_Sn(5tpO) **2**.

	1	2
Empirical formula	C_8_H_12_N_4_O_1_Sn	C_17_H_30_N_4_O_1_Sn
Formula weight	298.90	425.16
Temperature (K)	300	200
Crystal system	monoclinic	monoclinic
Space group	P2_1_	C2/C
Z	2	8
a (Å)	6.9546(7)	26.570(4)
b (Å)	11.0787(9)	11.6290(4)
c (Å)	15.539(1)	17.403(2)
α (deg)	90	90
β (deg)	91.205(9)	131.45(2)
γ (deg)	90	90
Volume (Å^3^)	1196.9(2)	4030.5(9)
ρ_calc_ (g/cm^3^)	1.659	1.401
μ (mm^−1^)	2.212	1.277
Measured reflections	6727	8816
Independent reflections	4070	4032
R_1_[on F_0_2, I > 2σ(I)]	0.0746	0.0331
wR_2_ (all data)	0.2409	0.0615
